# Effects of *Astragalus membranaceus* roots supplementation on growth performance, serum antioxidant and immune response in finishing lambs

**DOI:** 10.5713/ajas.19.0295

**Published:** 2019-08-23

**Authors:** Xiaoyan Hao, Pengju Wang, Youshe Ren, Gentang Liu, Jianxin Zhang, Brian Leury, Chunxiang Zhang

**Affiliations:** 1 College of Animal Science and Veterinary, Shanxi Agricultural University, Taigu 030801, China; 2 Shanxi Collaborative Innovation Center for High-Productive and Safe Livestock, Taigu 030801, China; 3 Institution of Husbandry and Veterinary, Yingxian 037600, China; 4 Faculty of Veterinary and Agricultural Sciences, University of Melbourne, Victoria 3010, Australia

**Keywords:** *Astragalus membranaceus* Root, Growth Performance, Antioxidant Status, Immune Response, Lamb

## Abstract

**Objective:**

*Astragalus membranaceus* root is a well-known traditional Chinese herbal medicine with many biological active constituents. This study was conducted to examine the effects of *Astragalus membranaceus* root powder (AMP) on growth performance, serum antioxidant and immune response in finishing lambs.

**Methods:**

A total of thirty-six Guangling fat-tailed ram lambs (body weight = 19±2 kg, mean ±standard deviation) were randomly assigned to one of six treatments for a 40 d feeding period, with the first 10 d for adaptation. Treatments consisted of the lambs’ basal diets with addition of 0, 5, 10, 15, 20, and 30 g/kg of diet of AMP.

**Results:**

Response to supplementation level of AMP was quadratic (p≤0.032) for final weight and ADG with the greatest at 10 g/kg of diet, but dry matter intake was not affected (p≥ 0.227) by treatments. The increase of AMP supplementation resulted in a quadratic response in contents of triglyceride and creatinine (p<0.05), with the lowest values for 10 and 20 g/kg of diet, respectively. A linear and quadratic decrease was observed in activity of alkaline phosphatase in serum of lambs. As the AMP supplementation increased, the activities of total superoxide dismutase and total antioxidant capacity increased linearly (p≤0.018) and hydroxyl radical (OH^−^) decreased linearly (p = 0.002). For catalase (CAT) and malondialdehyde (MDA), quadratic (p≤0.001) effects were observed among treatments, with the greatest CAT and lowest MDA values at 10 g/kg AMP. Additionally, supplementing AMP up to a level of 10 or 15 g/kg of diet quadratically increased immunoglobulin and interleukin contents in the serum.

**Conclusion:**

The results indicated that AMP can be used as natural feed additive in the ration of lambs to improve ADG, antioxidant status, and immune functions, and the optimal dose was 10 g/kg of diet under the condition of this experiment.

## INTRODUCTION

Over the past few decades, antibiotics have been used as animal growth promoters and antimicrobial drugs in livestock production. However, the use of antibiotics as growth promoters has been banned in many countries due to the great threat to food safety. Due to the high demand for organic animal products in the consumer market, attempts have been made to improve growth performance and immune function of finishing lambs by nutritional strategies instead of drugs enhancing performance. In recent years, different kinds of natural herbal plants, and/or their extracts, has been used as feed additives for animals due to their antimicrobial activities, immune enhancement and stress reduction properties [1,2].

The dried roots of *Astragalus membranaceus* (AM), also known as Huangqi, is a Chinese herbal medicine used in China for more than two thousand years, which contains more than 100 bioactive constituents, such as 24 polysaccharides, 11 monosaccharides, 13 triterpenes and flavonoids, 9 isoflavonoids, 25 amino acids, 21 inorganic elements, and γ-aminobutyric [3–6]. Previous studies have proved that AM roots possess growth-promoting, immunomodulatory, anti-oxidant, anti-bacterial, anti-viral and anti-inflammatory activities [7,8]. However, most of the research intensively focused on the physiological functions of AM or astragalus polysaccharide (APS) in humans and monogastric animals [9,10]. Available research data in terms of using AM to improve ruminant health are less common. Under intensive feeding conditions, the oxidative stress of fattening sheep increased. If AM can improve the immune function and antioxidant function of fattening sheep, it will be of great significance to the sheepmeat industry.

The objective of this study was to determine effects of dietary AM roots supplementation on growth performance, serum metabolites, antioxidant status, and immune responses of finishing lambs.

## MATERIALS AND METHODS

### Preparation and determination of the *Astragalus membranaceus* root powder

The sun-dried roots of 3-year-old AM were purchased from Yingxian Qianbao Co. Ltd (Shanxi, China), dried at 65°C for 3 h in electric thermostatic drying oven (Shantou Kehua Experimental equipment Co. Ltd., Shantou, China), and then ground to pass through a 2-mm screen to be an *Astragalus membranaceus* powder (AMP). The AMP was stored at ambient temperature (21°C to 24°C) before being mixed into the diets. The total polysaccharides were measured according to description of Yang et al [11]. The astragalosides and flavonoids were determined by high performance liquid chromatography analysis according to the method of Peng et al [5]. The chemical composition of AMP was shown in Table 1.

### Animals, diets, and experimental design

The use of the animals was approved by the Animal Care Committee, Shanxi Agricultural University (Taigu, China), and experimental procedures used in this study were in accordance with the university’s guidelines for animal research.

The experiment was arranged as a complete randomized design. Thirty-six Guangling fat-tailed ram lambs with similar weights (body weight [BW] = 19±2 kg) were randomly divided into six groups and allocated to 1 of 6 treatments. The treatments were: basal diet (control) and basal diet supplemented with AMP at the levels of 5, 10, 15, 20, and 30 g/kg of diet (treatments denoted as AMP5, AMP10, AMP15, and AMP20, and AMP30, respectively) by replacing equivalent amounts of corn straw in the diet formulation.

Diets were formulated to meet nutrient requirements of the animals [12]. Roughage and concentrate in diets were pelleted using a horizontal feed mixer (9SJW-300; National Science Makoto Farming Equipment Co. Ltd., Beijing, China), and the AMP was mixed during pelleting. The ration ingredient composition and nutrient levels were shown in Table 1. The experiment was conducted over 40 d, with the first 10 d for adaptation. Lambs were housed in individual stalls (3.0 m×0.8 m) and fed twice daily (07:00 and 17:00 h) at 105% of *ad libitum* intake and had free access to drinking water.

### Sample collection and analysis

During the experimental period, feed intake and refusal were recorded daily, and BW was weighed before the morning feeding for each lamb during a 3-d period at the start and end of experiment to determine dry matter intake (DMI), average daily gain (ADG), and feed conversion ratio. The contents of dry matter (DM) and crude protein, calcium, and phosphorus in diets were determined according to the method 930.15, 976.05, 927.02, 965.17 in official methods of analysis of the AOAC [13]. Neutral detergent fiber and acid detergent fiber content was analyzed using the Ankom A200 fiber analyzer (Ankom Technology, Macedon, NY, USA) by the method of Van Soest et al [14].

Blood samples were collected from the jugular vein using 10 mL evacuated blood-collection tubes before the morning feeding on the last day of the experiment period. Blood was centrifuged at 3,000 r/min for 15 min and serum was collected and stored at −20°C before analysis for total antioxidant capacity (T-AOC), malondialdehyde (MDA), hydroxyl radical inhibition capability (OH^−^), activities of glutathione peroxidase (GSH-Px), and total superoxide dismutase (T-SOD). These indicators were determined by colorimetry using standard commercial kits (Beijing Huaying Bioengineering Institute, Beijing, China) according to the manufacturer’s instructions. The content of total protein, globulin (GLO), albumin (ALB), blood urea nitrogen (BUN), triglyceride (TG), total cholesterol (TC), high density lipoprotein cholesterol (HDLC), low density lipoprotein cholesterol (LDLC), glucose (GLU), creatinine (Cr), activity of alanine aminotransferase (ALT), aspartate aminotransferase (AST) and alkaline phosphatase were determined using a fully automatic biochemistry analyzer (Shenzhen Mindrary Bio-Medical Electronics Co. Ltd. Shenzhen, China). The content of nitric oxide (NO) was determined by nitric reductase method using commercial kits (Shanghai Ruiqi Bioengineering Institute, Shanghai, China). The levels of immunoglobulin A (IgA), IgM, IgE, interleukin-2 (IL-2), IL-6, lysozyme (LZM), nitric oxide synthase (NOS), alexin C3 and C4, and tumor necrosis factor-α were determined by enzyme-linked immunosorbent assay commercial kits (Wuhan Boster Bio-engineering Limited Company, Wuhan, China).

### Statistical analysis

Data were analyzed statistically by one-way analysis of variance using the general linear model procedures of SAS (version 9.1, SAS Institute Inc., Cary, NC, USA), where dietary concentration of AMP as the treatment effect. Each sheep was defined as the experimental unit. In addition, the linear and quadratic effects of treatment were tested by an orthogonal polynomial contrast. Means were considered significantly different at p<0.05, and tendencies for treatment effects were defined as p<0.10.

## RESULTS

### Feed intakes and lamb performances

The DMI and growth performance are shown in Table 2. The initial BW of lambs was similar, but supplementation of AMP up to a level of 10 g/kg of diet quadratically increased final weight. The DMI was not affected by treatment (p = 0.227). The addition of AMP also displayed a quadratic response on the ADG (p = 0.032) and DMI/ADG ratio, with the greatest and lowest values for AMP10 diet (261.0 g/d and 3.98, respectively).

### Serum metabolites and indices of liver and renal function

The increase of AMP supplementation in the ration resulted in a quadratic response in contents of TG and creatinine (p< 0.05; Table 3), and the values of 10 to 20 g/kg were lower. A linear decrease was observed in activity of ALP in serum of lambs (p = 0.0001). Nearly quadratic effect in concentration of GLO and GLU were also observed (p = 0.069 and p = 0.063). But the contents of total protein, ALB, TC, HDLC, LDLC, BUN, AST, and ALT in serum was not affected by AMP supplementation (p>0.05).

### Antioxidant function

As shown in Table 4, no significant effect of AMP supplementation was detected on GSH-Px activity in serum (p = 0.406). Supplementing AMP linearly promoted T-SOD activity (p = 0.018) and linearly declined OH− concentration (p = 0.002). The MDA contents decreased as the AMP supplementation increased in the diet, and both the linear and quadratic effects were significant (p = 0.001), with lowest value for AMP10. Additionally, feeding of AMP displayed a quadratic (p = 0.040) response on the CAT activity, with the greatest value for AMP5 and AMP10. T-AOC was linearly (p = 0.043) increased by the addition of AMP to the diet.

### Immune function

The results of immune function are listed in Table 5. Supplementing AMP up to a level of 10 or 15 g/kg of diet quadratically increased IgA (p = 0.037), IgM (p<0.0001), and IgG (p = 0.0003) contents in the serum. There were also quadratic effects on IL-2 and IL-6, with the greatest values at AMP5 and AMP10. The concentrations of alexin C3 and C4, NO, LZM, and NOS activity were not affected (p>0.101) by the addition of AMP to the diet.

## DISCUSSION

The AM is a type of traditional Chinese herbal medicine, which grows wildly or semi-wildly in Yingxian and Hunyuan county, Shanxi province, China. It has been reported that AM root produced in Shanxi has a good quality, which contains 1.49 g/kg total isoflavonoids, 2.12 g/kg total astragaloside, 13.48 g/kg total polysaccharides, 30.3 g/kg total amino acids, 0.57g/kg γ-aminobutyric acid [3]. Bioactive ingredients especially are rich in 3-year-old AM root. The AMP used in our study contained total polysaccharides 16.92 mg/g AMP, astragalosidesI-IV 1.74 mg/g AMP, four flavonoids 1.38 mg/g (Table 1), which have many biological activities, such as a growth-promoting function, immunomodulatory activity, antioxidant activity, and so on [15].

In recent years, there have been many successful applications of AMP in mono-gastric animals. Some researchers have shown that AMP supplementation accelerates weight gain in broilers [9], increases the laying rate and egg mass and improves the quality of laying hen [10]. However, limited studies to date have examined effects of feeding AMP on DM intake and growth performance in ruminants. In the present study, the growth performance data showed that all dietary treatments did not affect DMI and only AMP supplemented at the level of 10 g/kg increased ADG of lambs. Likewise, the improvement in feed conversion was mainly due to the increased ADG rather than the effect on feed intake. This indicates that AMP supplemented at the level of 10 g/kg diet has growth-promoting effect on finishing lambs. However, the growth-promoting effect of AMP on finishing lambs was not linear. This illustrated that AMP can promote growth of lambs at appropriate level, and that more is not better. This may be due to the adverse effects of high doses of AMP on rumen microorganisms, which need further study. In contrast to our results, Zhong et al [2] reported that AM root supplementation had no effects on feed intake, ADG, feed efficiency and apparent digestibility of nutrients. Differences may be due to the higher dose level (50 g/d) of AMP in their study. Because our results also showed a quadratic effect on growth performance, and high dose AMP did not increase ADG. Zhong et al [2] also reported that AM root supplementation increased the concentration of volatile fatty acids and ammonia nitrogen in the rumen, which provided more energy and nitrogen sources for animals and rumen microorganisms, and ultimately was conducive to the growth of lambs. Additionally, previous studies have reported that *Astragalus* root contains varieties of chemical compounds which have varying pharmaceutical and biological activities, such as antioxidant, antimicrobial, and immune-enhancing functions [3,16]. However, some components such as saponin and polyphenolics may have anti-nutritional effects [17,18]. Therefore, adding high AMP to the diet may have negative effects on nutrients digestion and absorption. Besides, as a kind of herbal medicine, AM may not be added to animal diets for too long. In most studies on the application effect of Chinese herbal medicine in animal production, the experimental period was usually set at 30 to 40 days [2,19,20]. In present study, we determined the growth performance of lambs added AMP for 40 days. Moreover, it is necessary to further study whether the long-term addition of AMP has negative effects on animals.

Prior works have documented that polysaccharides and flavonoid glycosides extracted from AM root plays an essential role in regulation of glucose and lipid metabolism [6,21]. In our study serum TG and GLU content quadratically decreased with the increase of the AMP supplementation, which is consistent with those of Zou et al [22] and Chen et al [23], finding that administration of polysaccharides of AM could improve glucose uptake in rats and decrease the blood TG and promote the lipid metabolism in diabetic hamster.

Elevation of serum ALT, AST, and ALP activity indicates the abnormality of liver function [24]. And the increase of creatinine concentration in serum results in renal injury [25]. The linear and quadratic decrease of ALP and the quadratic reduction of creatinine suggested that the addition of AMP in the ration of finishing lambs improved the liver function and renal function in this study. This is in line with the observation of Zhang et al [26], who found that the polysaccharides of AM improved the renal function in diabetic rats. When the cells receive stimuli, such as oxidative stress, nuclear factor-κB is released from inhibitory protein compound (IκB) to activate the gene expression of inflammatory cytokines and proliferation, which might contribute to the liver and renal damage. Nevertheless, APS could improve kidney function by reducing the mRNA level of nuclear factor-κB and raising the IκB mRNA expression [26]. Sun et al [27] also found the hepato-protective activity of AM might be associated its ability of scavenge free radicals and antioxidant. However, when the supplementation level of AMP was up to 30 g/kg for 30 d, serum creatinine concentration rose again in our study, which inferred that 30 g/kg supplementation of AMP in the long term may have an adverse effect on kidney.

The increased activities of T-SOD, CAT, and T-AOC but reduced OH^−^ and MDA concentration in the serum with AMP supplementation in this study indicated that AMP enhanced the antioxidant status of finishing lamb. This is consistent with the observation of Zhong et al [2], who also reported that increased activities of T-AOC, T-SOD, and CAT in the serum of weaned lambs resulted from dietary AMP supplementation at levels of 50 g/kg. It is well known that GSH-Px, T-SOD, and CAT are endogenous antioxidant enzymes which constitute the antioxidant defense enzymatic system. Therefore, the higher activities of these enzymes in the AMP-treatments in this study may have resulted in a greater capacity of lambs to scavenge free radicals and reactive oxygen species and reduce the MDA concentration, as indicated by the lower extent of lipid peroxidation. Additionally, the linearly increase of serum antioxidant status with AMP supplementation was due to the combined effect of antioxidant compounds in AMP. It is reported that there are varieties of naturally biological compounds (such as polysaccharides, saponins, and flavonoids, etc.) in AM, which have strong antioxidant activities [28,29]. These exogenous antioxidants can improve the antioxidant status by increasing endogenous antioxidants and scavenging free radical to some extent [27,30]. Our results show that supplement AMP exhibited antioxidant effects in finishing lambs, indicated that AMP might help alleviate the oxidative stress of lambs during high density finishing period in production.

As we all know, the immune state of the body is influenced by the degree of oxidative stress and the intake of antioxidants [31]. Exogenous antioxidants can alleviate oxidative stress by reducing lipid peroxidation and scavenging free radicals, and ultimately play an immunopotentiating role. Previous studies reported that polysaccharide fractions in AMP have extensive effects on alleviating immune stress and activating the immune system [20,32]. *Astragalus* polysaccharide can modulate humoral and cellular immune response, including increasing the production of antigen-specific antibody, activating B cells and macrophages, promoting T cell proliferation and regulating cytokine productions [33,34]. In our study, AMP supplemented at the level of 10 and 15 g/kg diet did affect immune responses of lambs, including inducing IgA, IgG, and IgM productions, and when the AMP supplemented at the level of 5 and 10 g/kg diet, the concentrations of IL-2 and IL-6 in serum were increased. These responses may protect lambs against pathogenic and nonpathogenic immune challenges. Therefore, we suspect that the immunomodulatory effect of AMP is partly due to the polysaccharide fractions (169.2 mg/10 g AMP, Table 1) in present study. However, Zhong et al [2] showed that supplementation with astragalus polysaccharide at 15 g/kg diet did not affect the immune responses of lambs and the possible reason may be that the supplemented dose was suboptimal. Additionally, Mao et al [20] found that saponin and beta-glucan in AMP have strong immune-enhancing effects in weaned pigs. In other words, there are many bioactive ingredients in AMP which have potent immune modulation properties. Therefore, future studies need to determine the mechanism of functional constituents in regulating immune function for lambs.

## CONCLUSION

Results from this study show that supplementing the diets of finishing lambs with AMP at the level of 10 g/kg increased growth performance without affecting DMI. Supplementation of AMP enhanced antioxidant status and retarded lipid oxidation in the serum. Additionally, AMP possessed immunomodulating effect at 10 and 15 g/kg diet by improving serum immunoglobulin and interleukin. However, when the dosage exceeded 20 g/kg, the effect was not significant. Consequently, we infer that AMP can be used as a natural feed additive in the finishing lambs, and the optimal dose was 10 g/kg under the condition of this study.

## IMPLICATIONS

*Astragalus membranaceus* root, a well-known Chinese herbal medicine, is used as a potential antioxidant and immunomodulating agent in Chinese medicinal prescriptions. Supplementing *Astragalus membranaceus* root up to a level of 10 g/kg of diet promotes the growth performance, and improves antioxidant status and immune functions in finishing lambs. However, when the dosage exceeded 20 g/kg, the effect was not obvious. Therefore, *Astragalus membranaceus* root will be as a safe feed additive in the ration of finishing lambs in sheep production.

## Figures and Tables

**Table 1:** Ingredients and chemical compositions of experimental diets and active ingredients of *Astragalus membranaceus*

Item	Content (% of DM)	*Astragalus membranaceus*	Content (mg/g)
	
Ingredients		Total polysaccharides	16.92
Corn stalk	32.87	Saponins	
Sunflower seed hull	10.32	Astragaloside I	0.62
Corn	29.68	Astragaloside II	0.41
Soybean meal	9.92	Astragaloside III	0.19
Linseed meal	14.97	Astragaloside IV	0.52
Salt	0.60	Total	1.74
Calcium hydrophosphate	0.56	Flavonoids	
Sodium bicarbonate	0.20	Calycosin - 7 - glycosides	0.23
Premix1)	0.90	Calycosin	0.29
Chemical compositions		Ononin	0.05
ME2) (MJ/kg DM)	9.62	Formononetin	0.81
Crude protein	11.7	Total	1.38
Neutral detergent fiber	34.3		
Acid detergent fiber	22.2		
Calcium	0.62		
Phosphorus	0.43		

AM, *Astragalus membranaceus*; DM, dry matter; ME, metabolizable energy.

1) Premix containing: 40 mg/kg of Fe, 20 mg/kg of Mn, 40 mg/kg of Zn, 15 mg/kg of Cu, 0.2 mg/kg of I, 0.2 mg/kg of Co, 0.2 mg/kg of Se.

2) ME was a calculated value, while the others were measured values.

**Table 2:** Effects of *Astragalus membranaceus* roots on dry matter intake and growth performance of ram lambs

Items	Dietary treatment1)						SEM	Contrast p-value2)	
	
	Control	AMP5	AMP10	AMP15	AMP20	AMP30		Linear	Quadratic
Initial weight (kg)	19.5	19.5	19.5	20.4	19.8	19.5	0.56	0.175	0.258
Final weight (kg)	25.7^ab^	25.9^ab^	27.3^a^	25.8^ab^	25.2^ab^	24.4^b^	1.06	0.737	0.050
ADG (g/d)	210.8^ab^	212.7^ab^	261.0^a^	179.7^b^	181.4^b^	180.3^b^	25.34	0.560	0.032
DMI (g/d)	957.0	922.8	1,021.9	915.9	866.3	868.3	73.48	0.392	0.227
DMI/ADG	4.60^ab^	4.41^ab^	3.98^b^	4.81^a^	4.78^a^	4.85^a^	0.26	0.860	0.050

AMP, *Astragalus membranaceus* root powder; SEM, standard error of the mean; ADG, average daily gain; DMI, dry matter intake.

1) Control, AMP5, AMP10, AMP15, AMP20, and AMP30 were ram lambs fed the basal diet and the basal diet supplemented with AMP at a concentration of 5, 10, 15, 20, or 30 g/kg, respectively.

2) Dose response (linear or quadratic) to different levels of AMP supplementation.

ab Means within a row with different superscripts differ (p<0.05).

**Table 3:** Effects of the *Astragalus membranaceus* roots supplementation on serum biochemical index and enzymes related to liver function of ram lambs

Items	Dietary treatment1)						SEM	Contrast p-value2)	
	
	Control	AMP5	AMP10	AMP15	AMP20	AMP30		Linear	Quadratic
Total protein (g/L)	66.38	68.32	69.64	69.00	69.00	65.52	0.72	0.657	0.114
ALB (g/L)	28.24	28.04	29.06	28.36	28.03	28.92	0.25	0.591	0.846
GLO (g/L)	38.14	40.28	40.58	40.64	40.96	36.50	0.69	0.510	0.069
BUN (mmoL/L)	8.66	9.46	9.68	10.00	9.45	9.24	0.19	0.551	0.178
TC (mmol/L)	1.46	1.39	1.42	1.62	1.31	1.34	0.04	0.468	0.635
TG (mmol/L)	0.50^a^	0.40^ab^	0.35^b^	0.38^ab^	0.32^b^	0.40^ab^	0.01	0.149	0.023
HDLC (mg/dL)	0.98	0.93	1.06	1.05	0.85	0.98	0.03	0.678	0.907
LDLC (mg/dL)	0.47	0.44	0.44	0.53	0.42	0.42	0.02	0.563	0.695
GLU (mmol/L)	2.49	1.91	1.83	2.09	1.88	1.91	0.08	0.09	0.063
Cr (μmol/L)	50.58^bc^	51.18^b^	49.10^bc^	45.23^bc^	44.03^c^	57.63^a^	1.12	0.097	0.012
ALT (IU/L)	13.5	11.96	14.56	13.14	13.73	8.28	0.87	0.159	0.122
AST (IU/L)	101.8	95.7	111.2	105.6	115.3	109.5	3.25	0.201	0.364
ALP (IU/L)	407.3^a^	390.6^a^	409.5^a^	344.5^ab^	256.8^b^	247.7^b^	18.28	0.0001	0.136

AMP, *Astragalus membranaceus* root powder; SEM, standard error of the mean; ALB, albumin; GLO, globulin; BUN, urea nitrogen; TC, cholesterol; TG, triglyceride; HDLC, high density lipoprotein cholesterol; LDLC, low density lipoprotein cholesterol; GLU, glucose; Cr, creatinine; ALT, alanine aminotransferase; AST, aspartate aminotransferase; ALP, alkaline phosphatase.

1) Control, AMP5, AMP10, AMP15, AMP20, and AMP30 were ram lambs fed the basal diet and the basal diet supplemented with AMP at a concentration of 5, 10, 15, 20, or 30 g/kg, respectively.

2) Dose response (linear or quadratic) to different levels of AMP supplementation.

a–c Means within a row with different superscripts differ (p<0.05).

**Table 4:** Effects of the *Astragalus membranaceus* roots supplementation on antioxidant function of ram lambs

Items	Dietary treatment1)						SEM	Contrast p-value2)	
	
	Control	AMP5	AMP10	AMP15	AMP20	AMP30		Linear	Quadratic
GSH-Px (IU/mL)	807	849.1	845.5	842.5	800.7	792	27.15	0.406	0.425
T-SOD (IU/mL)	70.0^b^	79.2^b^	83.8^ab^	81.0^ab^	88.2^a^	77.2^b^	4.74	0.018	0.091
OH^−^ (IU/mL)	702.7^a^	686.0^ab^	648.6^bc^	646.2^bc^	620.6^c^	602.2^c^	25.73	0.002	0.765
CAT (IU/mL)	46.0^b^	51.3^a^	52.9^a^	47.3^ab^	47.8^ab^	47.6^ab^	2.14	0.869	0.040
MDA (nmol/mL)	5.34^a^	4.09^b^	3.18^c^	3.45^b^	4.07^b^	4.33^b^	0.378	0.001	0.001
T-AOC (IU/mL)	5.95^b^	6.42^ab^	6.36^ab^	6.48^ab^	6.97^a^	7.33^a^	0.458	0.043	0.481

AMP, *Astragalus membranaceus* root powder; SEM, standard error of the mean; GSH-Px, glutathione peroxidase; T-SOD, total superoxide dismutase; OH^−^, hydroxyl radical inhibition capability; CAT, catalase; MDA, malondialdehyde; T-AOC, total antioxidant capacity.

1) Control, AMP5, AMP10, AMP15, AMP20, and AMP30 were ram lambs fed the basal diet and the basal diet supplemented with AMP at a concentration of 5, 10, 15, 20, or 30 g/kg, respectively.

2) Dose response (linear or quadratic) to different levels of AMP supplementation.

a–c Means within a row with different superscripts differ (p<0.05).

**Table 5:** Effects of the *Astragalus membranaceus* roots on immune function of ram lambs

Items	Dietary treatment1)						SEM	Contrast p-value2)	
	
	Control	AMP5	AMP10	AMP15	AMP20	AMP30		Linear	Quadratic
IgA (g/L)	0.46^b^	0.49^b^	0.51^b^	0.60^a^	0.48^b^	0.50^b^	0.012	0.102	0.037
IgM (g/L)	1.05^b^	1.19^b^	1.52^a^	1.53^a^	1.11^b^	1.22^b^	0.040	0.399	<0.0001
IgG (g/L)	20.5^b^	22.6^b^	28.2^a^	29.0^a^	19.6^b^	21.5^b^	0.95	0.239	0.0003
IgE (IU/mL)	43.0	42.4	44.2	45.6	43.5	42.7	0.61	0.183	0.536
TNF (pg/mL)	63.5	62.0	61.8	63.3	62.4	61.7	0.87	0.929	0.523
IL-2 (ng/mL)	3.74^b^	6.26^a^	6.96^a^	3.13^b^	3.29^b^	2.39^b^	0.322	0.522	<0.0001
IL-6 (pg/mL)	106.9^b^	122.5^ab^	128.5^a^	113.9^ab^	112.8^ab^	105.4^b^	2.83	0.360	0.031
Alexin C3 (g/L)	0.48	0.43	0.38	0.47	0.42	0.40	0.021	0.717	0.146
Alexin C4 (g/L)	0.098	0.089	0.077	0.088	0.081	0.074	0.006	0.367	0.354
NO (μmol/L)	34.3	32.3	33.6	35.8	35.5	34.5	0.59	0.287	0.101
NOS (U/mL)	19.3	18.7	18.9	18.9	20.4	18.0	0.66	0.834	0.820
LZM (μg/mL)	21.0	22.6	22.1	23.1	22.4	20.6	0.37	0.309	0.401

AMP, *Astragalus membranaceus* root powder; SEM, standard error of the mean; Ig, immunoglobulin; TNF-α, tumor necrosis factor-α; IL, interleukin; NO, nitric oxide; NOS, nitric oxide synthase; LZM, lysozyme.

1) Control, AMP5, AMP10, AMP15, AMP20, and AMP30 were ram lambs fed the basal diet and the basal diet supplemented with AMP at a concentration of 5, 10, 15, 20, or 30 g/kg, respectively.

2) Dose response (linear or quadratic) to different levels of AMP supplementation.

ab Means within a row with different superscripts differ (p<0.05).
